# Effects of single and multiple transplantations of human umbilical cord mesenchymal stem cells on the recovery of ovarian function in the treatment of premature ovarian failure in mice

**DOI:** 10.1186/s13048-021-00871-4

**Published:** 2021-09-15

**Authors:** Xiaodan Lv, Chunyi Guan, Ying Li, Xing Su, Lu Zhang, Xueqin Wang, Hong-Fei Xia, Xu Ma

**Affiliations:** 1 Reproductive and Genetic Center of the National Research Institute for Family Planning, Beijing, 100081 China; 2 Graduate School, Peking Union Medical College, Beijing, 100730 China

**Keywords:** Premature ovarian failure, hUC-MSCs, Transplantation, Ovarian function

## Abstract

**Background:**

Currently, there is no effective treatment for premature ovarian failure (POF), and stem cell therapy is considered the most promising treatment. Human umbilical cord blood mesenchymal stem cells (hUC-MSCs) have shown good regenerative ability in various diseases, including POF; however, their underlying mechanism and dosage for POF treatment remain unclear. This study aimed to compare the effect of single and multiple injections of hUC-MSCs on ovarian function repair in chemotherapy-induced POF.

**Methods:**

Female mice were intraperitoneally injected with 30 mg/kg busulfan and 120 mg/kg cyclophosphamide (CTX) to induce POF. In the single hUC-MSC injection group, hUC-MSCs were transplanted into mice D7 after CTX and busulfan administration, while in the multiple injection group, hUC-MSCs were transplanted on D7, D14, and D21 after CTX and busulfan administration. We evaluated the ovarian morphology, fertility, follicle-stimulating hormone and estradiol concentrations, follicle count, POF model, and cell transplantation results. In addition, real-time polymerase chain reaction, immunohistochemistry, and miRNA and mRNA chips were used to evaluate the effect of the cell therapy.

**Results:**

Ovary size, number of follicle at all developmental stages, and fertility were significantly reduced in the POF group compared with the control. Under hUC-MSC treatment, the ovarian morphology and follicle count were significantly restored, and fertility was significantly increased. By comparing the single and multiple hUC-MSC injection groups, we found that the anti-Müllerian hormone and Ki-67 levels were significantly increased in the multiple hUC-MSC group on D60 after chemotherapy. The expression of stimulating hormone receptors, inhibin α, and inhibin β was significantly restored, and the therapeutic effect was superior to that of the single hUC-MSC injection group.

**Conclusion:**

These results indicate that hUC-MSCs can restore the structure of injured ovarian tissue and its function in chemotherapy-induced POF mice and ameliorate fertility. Multiple hUC-MSC transplantations have a better effect on the recovery of ovarian function than single hUC-MSC transplantation in POF.

## Background

Premature ovarian failure (POF) usually manifests as amenorrhea and infertility; the serum follicle-stimulating hormone (FSH) level is increased, and the levels of anti-Müllerian hormone (AMH) and serum estrogen are reduced [1]. Anticancer therapeutics, such as cyclophosphamide (CTX) and busulfan, have been shown to be highly damaging to ovarian follicles [2]. Therefore, the preservation of fertility and ovarian function should be the main considerations for chemotherapy in women of childbearing age. Recently, stem cells have been used to restore the normal functions of injured tissues or organs [3–5] and are also considered a novel option for treating female infertility [6–9].

Many studies on POF animal models have confirmed that the administration of MSCs obtained from various cell types can protect and possibly restore ovarian function and structure [10–12]. Among the various MSCs, human umbilical cord blood-derived MSCs (hUC-MSCs) have attracted much attention because of their low immunogenicity and potential use in allogeneic therapy [13]. Moreover, hUC-MSCs do not require invasive methods to obtain sufficient cells for use in regenerative therapy. Studies have shown that MSCs promote ovarian cell growth and improve ovarian function through paracrine effects [14]. Therefore, the use of secretins, cytokines, and exosomes derived from MSCs to develop cell-free therapies that can circumvent cellular immunogenicity [15, 16] has become the focus of an increasing number of studies. Gupta et al. used the secretome of human uterine cervical stem cells to treat menopausal women and restore their reproductive function [17], and Ahmadian et al. used platelet-rich plasma to restore fertility in a POF mouse model [18]. Although cell-free therapy has a positive therapeutic effect on POF, the paracrine effect of MSCs is not the main reason MSC therapy plays a role in POF treatment. Ling et al. found that the therapeutic effect of MSC medium on POF is not as good as that of MSCs [19]; therefore, exploring the quality and dosage of MSCs and their mechanism of action is still the focus for determining the therapeutic effect of MSCs [20].

However, the dose for hUC-MSC transplantation and its therapeutic effect on POF remains unclear. The purpose of this study was to investigate the therapeutic effect of hUC-MSCs on the ovarian function in chemotherapy-induced POF mice and to evaluate the effect of single and multiple injections of hUC-MSCs on ovarian function recovery.

## Materials and methods

### Isolation and culture of hUC-MSCs

Human umbilical cord samples were obtained from full-term babies delivered by cesarean section at the Haidian Maternal Child Health Hospital. The umbilical cords were washed with PBS to remove blood. The arteries and veins were removed from the umbilical cords, after which they were cut into small pieces of 0.5–1 mm^3^ and placed at the bottom of a tissue culture dish. The Petri dish was then placed in an incubator at 37 °C with 5% CO_2_. Cells were cultured in MEMα complete medium (10% fetal bovine serum (FBS), 100 U/mL penicillin G, and 0.1 mg/mL streptomycin). When the cells reached approximately 90% confluence, they were digested using 0.25% trypsin, and the 1:2 separation and passage was continued for the cells to reach the logarithmic growth phase [21, 22].

### Establishment of the POF mouse model and grouping

**Fig. 1:**
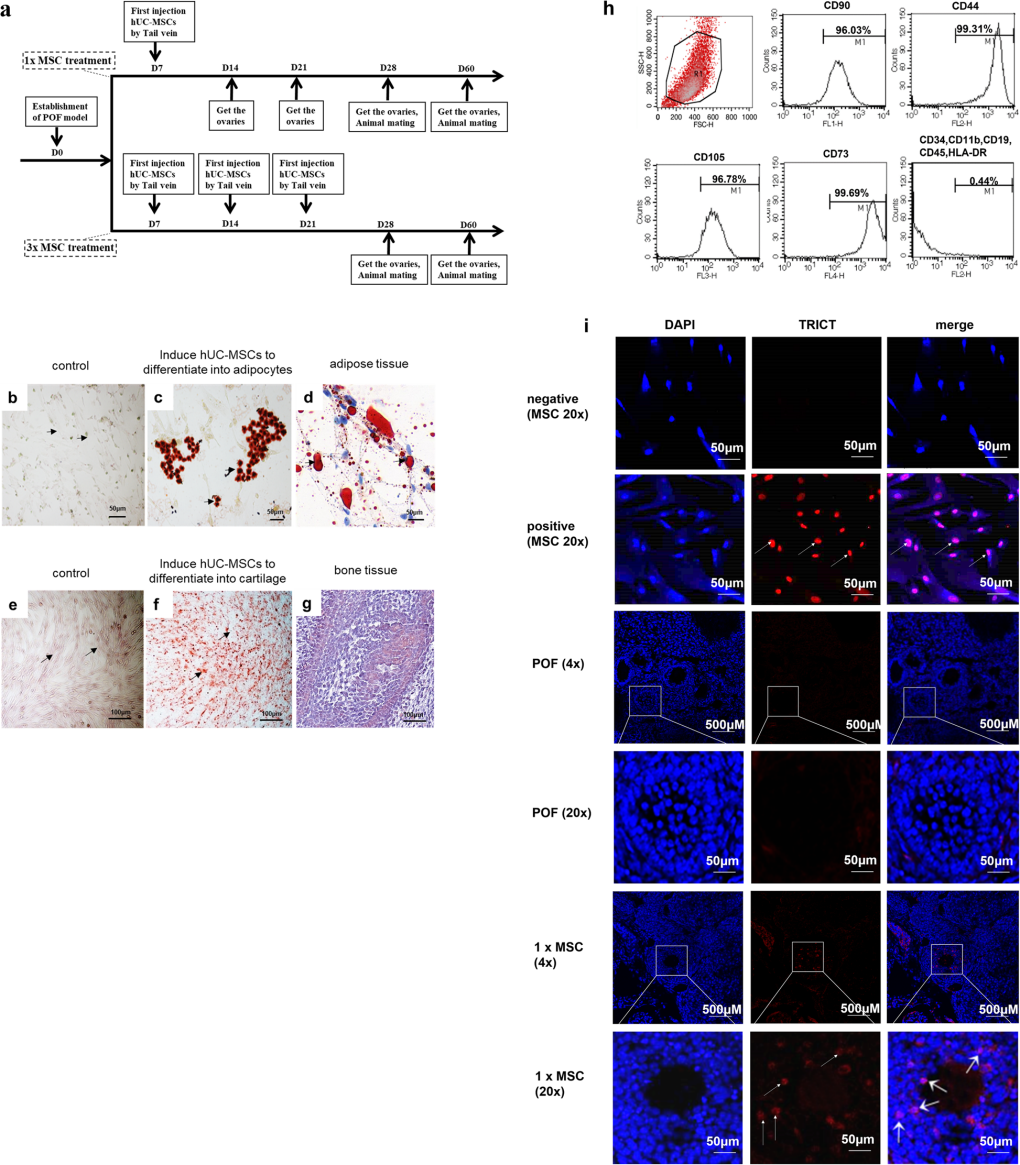
The characterization and differentiation of hUC-MSCs. **a** The experimental procedure of hUC-MSCs in the treatment of POF. **b**, **c**, **d** The hUC-MSCs differentiate into osteoblasts, scale bar = 50 μm. *n* = 3. **e**, **f**, **g** adipocytes, scale bar = 100 μm. *n* = 3. **h** Flow cytometric analysis of hUC-MSCs. CD44, CD73, CD90, and CD105 were positive and CD34, CD11b, CD19, CD45 and HLA-DR were negative. *n* = 3. **i** Human nuclei antigen was negative in POF murine ovarian section, the recipient ovaries after hUC-MSCs transplantation were expressed human nuclei antigen. Stain hUC-MSCs as a positive control. *n* = 3

Six-week-old female Institute of Cancer Research mice were purchased from Huayikang Biotechnology Co., Ltd, Beijing, China. All animals had free access to food and water. Vaginal smears were used to monitor the estrus cycle. As exhibited in Fig. 1a, the experiment consisted of two groups: single transplantation group (named 1 × MSC treatment) and thrice transplantation group (named 3 × MSC treatment). Overall, 200 mice were randomly divided into four sub-groups: sham, POF, and MSC treatment groups. The mice in the sham group were injected with saline only. The mice in the POF group were intraperitoneally injected with chemotherapeutic drugs (a mixture of 120 mg/kg CTX and 30 mg/kg busulfan) to establish a POF model. The day of chemotherapeutic drug administration was recorded as day 0 (D0). The mice in the MSC treatment group were given chemotherapeutic drugs, and then injected with 100 μL of 2 × 10^6^ hUC-MSC suspensions per transplantation by the tail vein [23]. In the 1 × MSC treatment group, hUC-MSCs were injected into the tail vein on D7 after chemotherapy; then 5 mice in each group were removed from the ovaries at D14, D21, D28 and D60 after chemotherapy, while 5 mice in each group were subjected to mating experiments for fertility testing. In the 3 × MSC treatment group, hUC-MSCs were injected into the tail vein on D7, D14, and D21 after chemotherapy, then the ovaries of 5 mice in each group were removed on D28 and D60 after chemotherapy, while 5 mice in each group were subjected to mating experiments for fertility testing. In the fertility testing experiment, the mice were caged at a 2:1 male to female ratio. All procedures were approved by the Animal Protection and Use Committee of the National Family Planning Society of China. This study was conducted in strict accordance with the recommendations in the “Guidelines for the Care and Use of Laboratory Animals of the National Institutes of Health.”

### Organ coefficient and pregnancy rate

The mice were injected with 10 IU of PMSG 2 d before euthanasia to ensure that the mice in each group were in the same estrus cycle. The mice were weighed before being euthanized, and the ovaries were quickly removed and weighed after euthanasia to calculate the organ coefficient as follows, using at least five mice per group:

$$Organ coefficient (\%)=\left(\frac{ovarian weight}{mouse weight}\right)\times 100$$

One ovary from each mouse was retrieved for tissue analysis and the other for molecular biology testing. An effective pregnancy was considered when the mice gave birth. The fertility rate was determined using the following formula, using at least five mice per group:

$$Pregnancy rate \left(\%\right)=\left(\frac{number of pregnant mice in each group}{number of mice in each group}\right)\times 100$$

### Immunophenotypic analysis

A Human MSC Analysis Kit (BD Biosciences, Franklin Lakes, NJ, USA) was used to identify the MSCs. Cells were resuspended in a 1 × 10^7^ cells/mL buffer, and 100 µL of prepared cell suspension was added to nine tubes and incubated with various fluorescent dye-conjugated specific antibodies, including FITC mouse anti-human CD90 (tube 1), PE mouse anti-human CD44 (tube 2), PerCP-Cy™5.5 mouse anti-human CD105 (tube 3), APC mouse anti-human CD73 (tube 4), nothing (tube 5), hMSC positive isotype control cocktail and PE hMSC negative isotype control cocktail (tubes 6 and 8, respectively), and hMSC positive and PE hMSC negative cocktails (tubes 7 and 9, respectively), in the dark for 30 min at 20 °C. The results were analyzed by flow cytometry, and the experimental results were verified thrice.

### Osteogenic differentiation

To promote osteogenic differentiation, the cells were seeded into 12-chamber slides and cultured in Dulbecco’s modified Eagle’s medium (HyClone Laboratories Inc., Logan, Utah, USA) containing 10% FBS. When the cells reached 70–80% confluence, the medium was replaced with osteogenic differentiation medium (HyClone Laboratories Inc.), which was changed every 3 d [24]. Two weeks later, the calcified extracellular matrix was stained with 2% Alizarin red to confirm osteogenic differentiation. The experiment was repeated thrice.

### Adipogenic differentiation

To induce adipogenic differentiation, cells were cultured in an MSC medium until they reached 90% confluence. The cells were then induced in an adipogenic induction medium (MEMα modification, HyClone Laboratories Inc.) for 3 d. Afterward, the adipogenic induction medium was replaced with a maintenance medium containing 10 µM insulin and MSC medium and changed every 3 d. For the negative control, cells were cultured in an MSC medium [25]. After 1 week, the intracellular lipid droplets formed were stained using Oil red O staining. The experiment was repeated thrice.

### ELISA detection of mice estradiol (E2) and follicle-stimulating hormone

Blood samples were collected on D14, D21, D28, and D60 after POF induction. The samples were incubated overnight at 4 °C and centrifuged at 4,000 rpm for 5 min, and the supernatant was collected. The levels of E_2_ and FSH were measured using the Mouse E_2_ ELISA Kit (Bio-Swamp, Wuhan, China) and Mouse FSH ELISA Kit (Bio-Swamp), respectively. Each experiment was repeated five times.

### Histologic staining and follicle counting

The ovaries were fixed overnight in 4% paraformaldehyde, embedded in paraffin by dehydration, and serial sectioning was performed. The thickness of each slice was 5 μm, with each one obtained every 20 μm. Three slices per mouse were used for hematoxylin and eosin (H&E) staining. The ovarian primordial, primary, secondary, and mature follicles were observed and counted under a TE2000-u reverse-phase microscope (Nikon, Tokyo, Japan). The number of follicles were counted at different points of the section, and the classification and characteristics of all stages of follicle development were determined as follows: primitive follicles were close to the white membrane, and the oocyte was surrounded by a single layer of flat granular cells; primary growth follicles were surrounded by one or more layers of cubic granular cells, and red staining appeared between the two zona pellucida; the follicular membrane of the connective tissue appeared on the periphery of the follicle, a follicular cavity appeared in secondary growth follicles, and the follicular membrane could be separated into inner and outer membranes; mature follicles had an obvious cumulus, large follicular cavity, and many capillaries between the cells; and atresia follicles had a collapsed follicle wall, unclear or sometimes absent egg cell structure, and a shrunken zona pellucida. At least five mice in each group were selected for statistical analysis.

### Immunohistochemical analysis

The paraffin sections were deparaffinized, rehydrated, and pressurized (high pressure) in a citrate buffer (pH 6.0) for 2 min to determine antigenicity. The samples were then incubated with 3% hydrogen peroxide for 10 min to quench endogenous peroxidase activity. Sections were blocked with non-specific antigens using 10% goat serum. Sections were then incubated with Anti-Ki67 antibody (1:100; Abcam, Cambridge, UK) or mouse anti-human AMH (1:30; Bio-Rad AbD Serotec Limited, Oxford, UK) in a humidified chamber at 4 °C overnight. Negative controls were incubated with 10% goat serum at 4 °C overnight. After incubation with the primary antibody, peroxidase-conjugated AffiniPure goat anti-rabbit IgG (1:200; ZSBIO, Beijing, China) or peroxidase-conjugated AffiniPure goat anti-mouse IgG (1:200; ZSBIO) was added for 1 h at room temperature. Subsequently, the sections were stained using 3, 3-diaminobenzidine (ZSBIO) at room temperature without light for 10 min and counterstained with hematoxylin for 10 s. After sealing the slides, the samples were photographed using a microscope (Nikon).

The number of Ki67-labeled proliferating cells and the total number of cells were counted in at least three high-power fields randomly selected for each slice, and at least 100 cells were counted. The proportion of proliferating cells was expressed as the ratio of the number of Ki67-positive cells to the total number of cells. At least three sections were observed for each sample. The positive signal intensity of AMH was analyzed using ImageJ software (NIH, Bethesda, Maryland) to measure the average optical density (MOD) [26]. In short, a 24-bit RGB image was first converted to an 8-bit grayscale image. The MOD percentage of the AMH signal was expressed as the ratio of the AMH positive staining area to the entire field of view area. Three fields of view were randomly selected for each slice, and three different slices were selected for the same sample. There were at least five samples in each group.

### Immunofluorescence staining

Ovary samples were collected and fixed in 4% paraformaldehyde at 4 °C overnight, and then dehydrated with 30% sucrose for 1 week. MSCs were fixed with 4% paraformaldehyde for 15 min. Samples were then fixed using the Tissue-Tek OCT Compound (Sakura Finetek Middle East, Dubai, UAE) at − 80 °C and sliced into 7 μm-thick sections at − 25 °C. Slides were blocked with 10% goat serum for 1 h at room temperature and then incubated with mouse anti-human nuclei monoclonal antibody (1:100; Millipore Sigma, Burlington, MA, USA) at 4 °C overnight. Goat serum (10%), the primary antibody, was used as a negative control. Sections were probed with TRITC-labeled IgG (1:200; ZSBIO) and counterstained using 4′ 6-diamidino-2-phenylindole. Fluorescence images were obtained using a fluorescence microscope (DMI3000; Leica, Heidelberg, Germany). The experiment was repeated thrice. To evaluate the antibody specificity, negative control staining was performed by replacing the primary antibody with normal goat serum. hUC-MSCs were used as the positive control for staining against human nuclear antigen (Fig. 1i).

### Microarray hybridization and data analysis

According to the manufacturer’s instructions, the miRNA easy Mini Kit (Qiagen GmbH, Hilden, Germany) was used to isolate total RNA from the ovaries of three mice each from the control, POF, and hUC-MSC groups (7 days after the first injection of hUC-MSCs). The miRNA microarray, including probe labeling, hybridization, hybridization image scanning, and initial data analysis, was performed by LC Sciences (LC Sciences, Houston, TX, USA) using the LC-miRHumanMouseRat_11.0_080411 array. The mRNA chip used was the OneArray chip (Tecenet, Chengdu, China). The cyclic local weighted regression method was used for normalization. A *t*-test was performed between the control and POF groups, POF and hUC-MSC groups, and the hUC-MSC and control groups. Afterwards, statistical analysis of the microarray data was performed.

### Quantitative reverse-transcription polymerase chain reaction (qRT-PCR)

The TRIzol (Invitrogen, Carlsbad, CA, USA) method was used to extract RNA from ovarian tissue. The RNA was extracted using a reverse transcription kit (TaKaRa Biomedical Technology Co., Ltd., Beijing, China), which reverse-transcribed cDNA for qRT-RNA detection. The ovarian samples of mice in each group at D14, D21, D28, and D60 after chemotherapy were selected for RNA extraction. For real-time PCR detection, three parallel replicate holes were set for each mouse, and five mouse samples were selected for each group. Three independent experiments were repeated. Statistical significance was set at *P* < 0.05. The primer sequences used are as follows: mRNA-FSHR-Forward, 5'-GAGGTGCAAGCCCAGATTTA-3'; mRNA-FSHR-Reverse, 5'-GAGGGACAAGCACGTAACTATT-3'; mRNA-INHIBINα-Forward, 5'-TCTGAACCAGAGGAGGAAGAT-3'; mRNA-INHIBINα-Reverse, 5'-GGGATGGCCGGAATACATAAG-3'; mRNA-INHIBINβ-Forward, 5'-AAGAAAGAGGTGGATGGAGATG-3'; mRNA-INHIBINβ-Reverse, 5'-CAGCATGAGGAAAGGTCTATGT-3'; mRNA-GAPDH-Forward, 5'-GGTGAAGGTCGGTGTGAACG-3'; mRNA-GAPDH-Reverse, 5'-CTCGCTCCTGGAAGATGGTG-3'.

### Statistical analysis

The unpaired Student’s *t*-test was used to determine the significance between two groups. One-way analysis of variance was used to determine significant differences among the groups. Prism6.0 software was used for illustrations.

## Results

### hUC-MSCs express specific surface antigens and have the potential for multilineage differentiation

hUC-MSCs have multilineage differentiation potential and can induce differentiation in adipocytes (Fig. 1b–d) and osteoblasts (Fig. 1e–g). The expression rates of hUC-MSC surface antigens CD29, CD73, CD90, and CD105 were higher than 95%, and those of CD11b, CD19, CD34, CD45, and HLA-DR were lower than 5% (Fig. 1h). Frozen mouse ovary sections were used to detect the migration of hUC-MSCs in the body. As shown in Fig. 1i, hUC-MSCs injected through the tail vein of the mice survived in the body and migrated to the injured site after 7 d. The hUC-MSCs displayed by red fluorescence were mainly distributed in the follicular granulosa cells in the hUC-MSC treatment group, indicating that hUC-MSCs can survive in mice and migrate to damaged parts to play a repair role.

### Effects of hUC-MSC transplantation on the organ coefficient and fertility of ovaries in POF mice

**Fig. 2:**
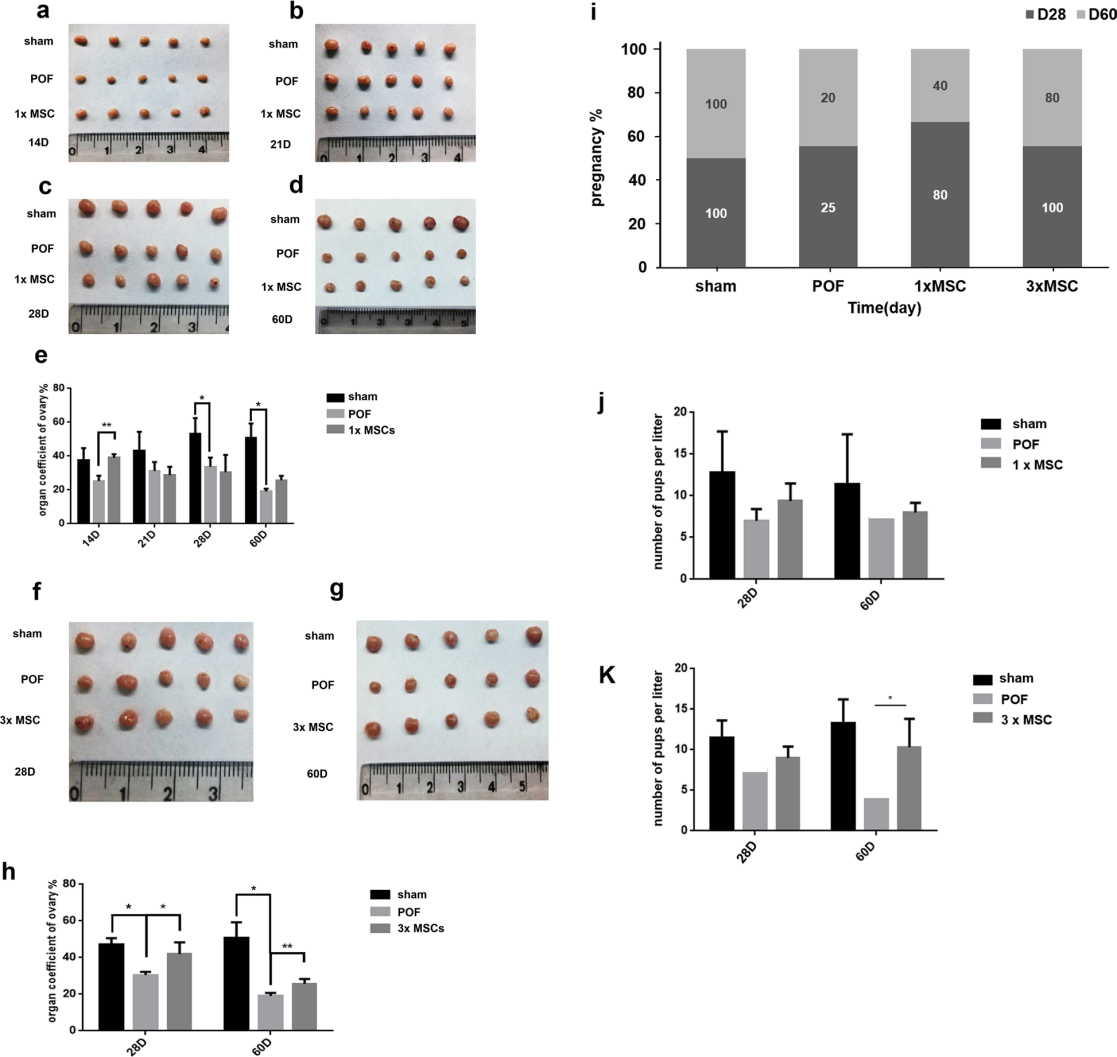
Changes of ovary weight in the once intravenous group and the multiple intravenous groups over 60 days. **a**, **b**, **c**, **d** In the once intravenous group, ovarian volume of mice was remarkably increased at 7, 14, 21, 28 days post-induction, respectively. *n* = 5. **f**, **g** The multiple intravenous groups showed significantly increased of ovarian volume at 28, 60 days post-induction, respectively. *n* = 5. **e**, **h** The histogram represents the results of statistical analysis of ovarian weight by single and multiple injections, respectively. Organ coefficient = organ weight/weight, *n* = 5. **i** Pregnancy rate of POF mice treated with D28 and D60, 1xMSC and 3xMSC. *n* = 5. **j** Number of litters per litter of POF treated with 1xMSC. *n* = 5. **k** Number of litters per litter of POF treated with 3xMSC. *n* = 5. *P < 0.05; **P < 0.01. 1xMSC: Single transplantation of hUC-MSC; 3xMSC: Multiple transplantation of hUC-MSC

The therapeutic effect of hUC-MSCs on POF was evaluated using the ovarian organ coefficient. On D28 and D60, the POF group showed a significant decrease in the organ coefficient ratio compared to the sham group (*P* < 0.05, Fig. 2). The organ coefficient ratio of the ovaries of the mice in the single hUC-MSC injection group was significantly increased on D14 compared with the POF group (*P* < 0.01, Fig. 2a–e). The organ coefficient of the mouse ovaries increased significantly on D28 and D60 in the multiple hUC-MSC injection group (*P* < 0.05, Fig. 2f–h) compared with the POF group. The results showed that the ovary organ coefficient was restored considerably after multiple hUC-MSC injections, which was superior to that after single hUC-MSC injection in treating POF. The mouse fertility test showed that both single and multiple hUC-MSC injections restored POF mouse pregnancy rate in the short- and long-term significantly (Fig. 2i). Figure 2j and k show the number of litters per litter of mice. Among them, the conception rate of mice in the 3 × MSC treatment group recovered more significantly at D60, and the number of litters per litter increased significantly compared with the POF group. The results showed that hUC-MSCs could significantly restore the ovarian function in POF mice.

### Transplantation of hUC-MSCs upregulates the ovarian reserve in POF mice

**Fig. 3:**
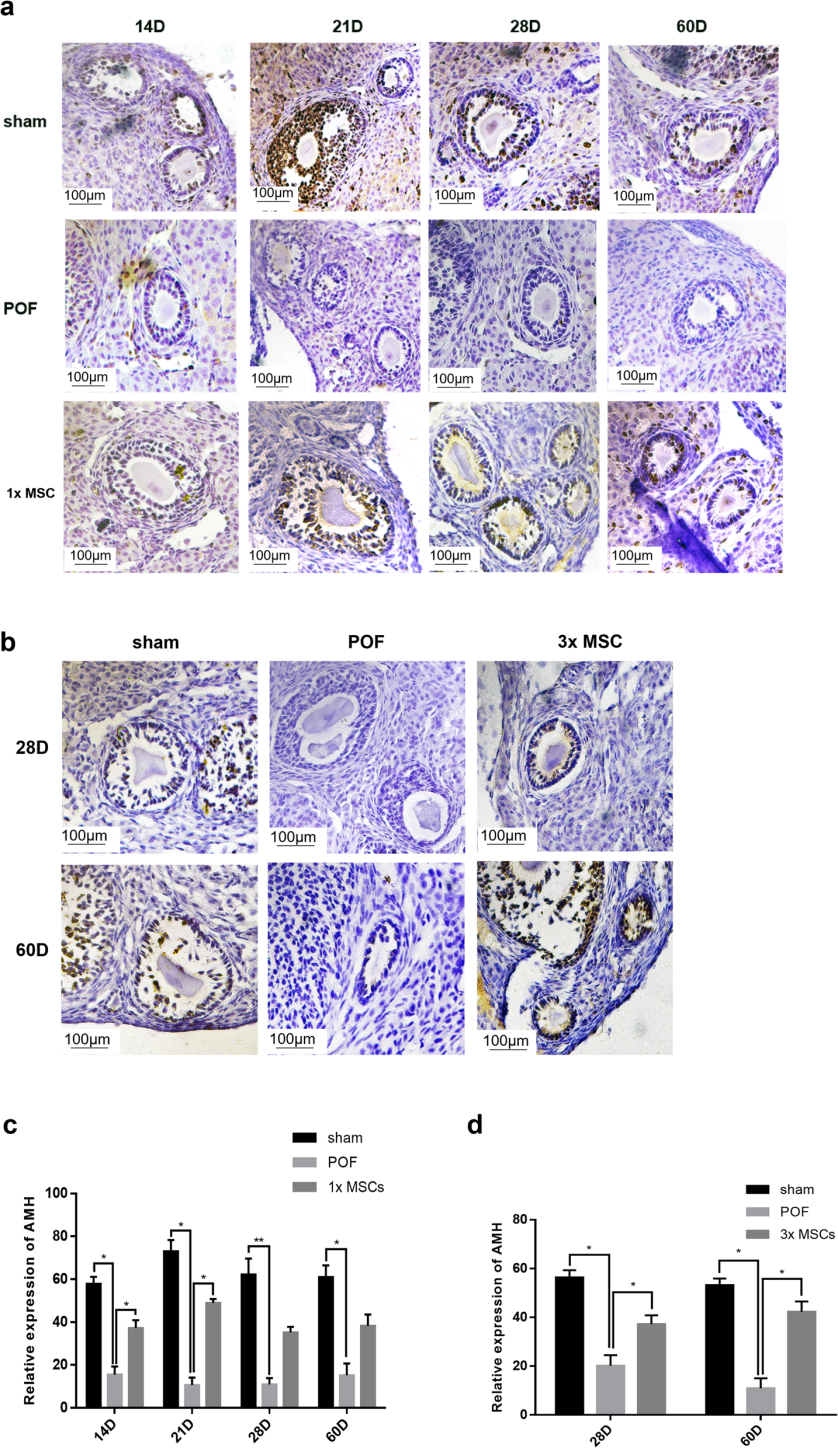
The ovarian reserve capacity analysis. Immunohistochemistry for AMH in once intravenous group (**a**, **c**) and the multiple intravenous group (**b**, **d**). Strong expression of AMH were seen in Sham group at each time point, granulosa cells were negative in POF group, AMH expression reappeared in ovaries from MSCs group. Original magnification: 400 × . *n* = 5. 1xMSC: Single transplantation of hUC-MSC; 3xMSC: Multiple transplantation of hUC-MSC

AMH was used to determine the mouse ovarian reserve. AMH is a member of the transforming growth factor β family and is expressed by granulosa cells. The level of AMH is significantly related to the reserve capacity of the ovaries. We performed immunohistochemical staining of the ovaries to determine AMH expression in the granulosa cells of antral follicles. The results are shown in Fig. 3. The POF group had significantly downregulated AMH expression compared with the sham group. On D14 and D21, AMH expression in the ovaries of the single hUC-MSC injection group was significantly upregulated compared with that of the POF group (Fig. 3a and c). On D28 and D60, AMH expression in the ovaries of the multiple hUC-MSC injection group was significantly upregulated compared with that of the POF group (Fig. 3b and d). The results showed that on D28 and D60, the recovery of ovarian reserve capacity after multiple hUC-MSC transplantation was superior to that of single hUC-MSC transplantation.

**Fig. 4:**
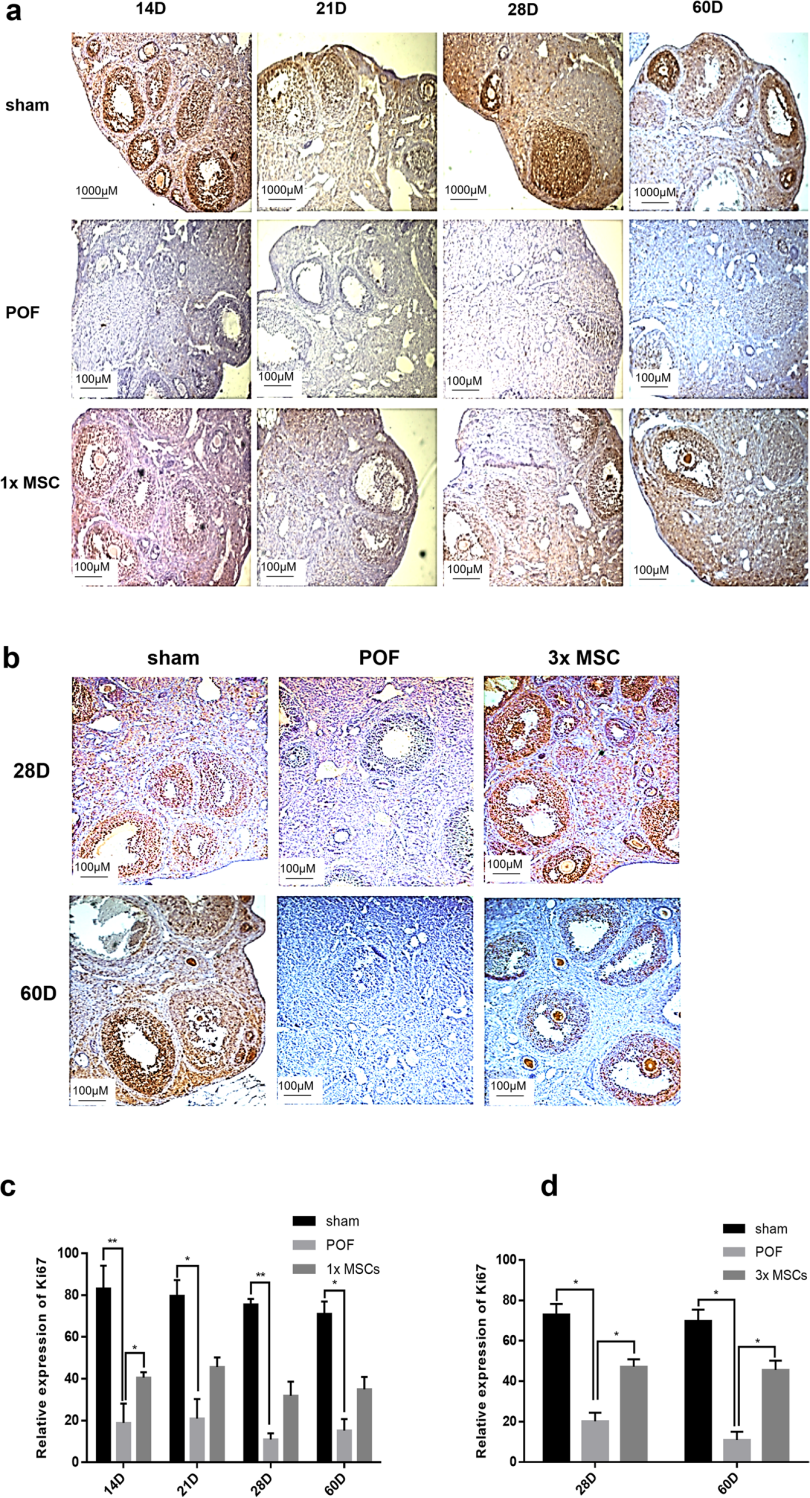
The comparison of ovarian proliferation potential in each group. Immunohistochemistry for KI67 in once intravenous group (**a**, **c**) and the multiple intravenous group (**b**, **d**). Sham mice follicle highly expressed KI67, POF mice follicle lacked KI67 signal, the expression was observed in MSCs group. Original magnification: 400 × . *n* = 5. 1xMSC: Single transplantation of hUC-MSC; 3xMSC: Multiple transplantation of hUC-MSC

Ki67 is a DNA-binding protein expressed in all active cell cycle stages and can be used as a marker for cell proliferation. The results of immunohistochemistry tests are shown in Fig. 4. The follicles of the POF mice were primarily negative, and the proliferation period was minimal compared with the sham group. In the single hUC-MSC injection group, the mouse follicles were slightly stained on D14, and the cell proliferation potential was restored (Fig. 4a and c). Deep mouse follicle staining was observed on D28 and D60 in the multiple hUC-MSC injection group relative to the POF group (Fig. 4b and d), indicating that the cell proliferation rate was significantly restored. The results showed that the multiple hUC-MSC transplantation group had a more significant recovery of the ovarian cell proliferation rate on D28 and D60 than the single hUC-MSC transplantation group.

### hUC-MSC transplantation restores follicular development

**Fig. 5:**
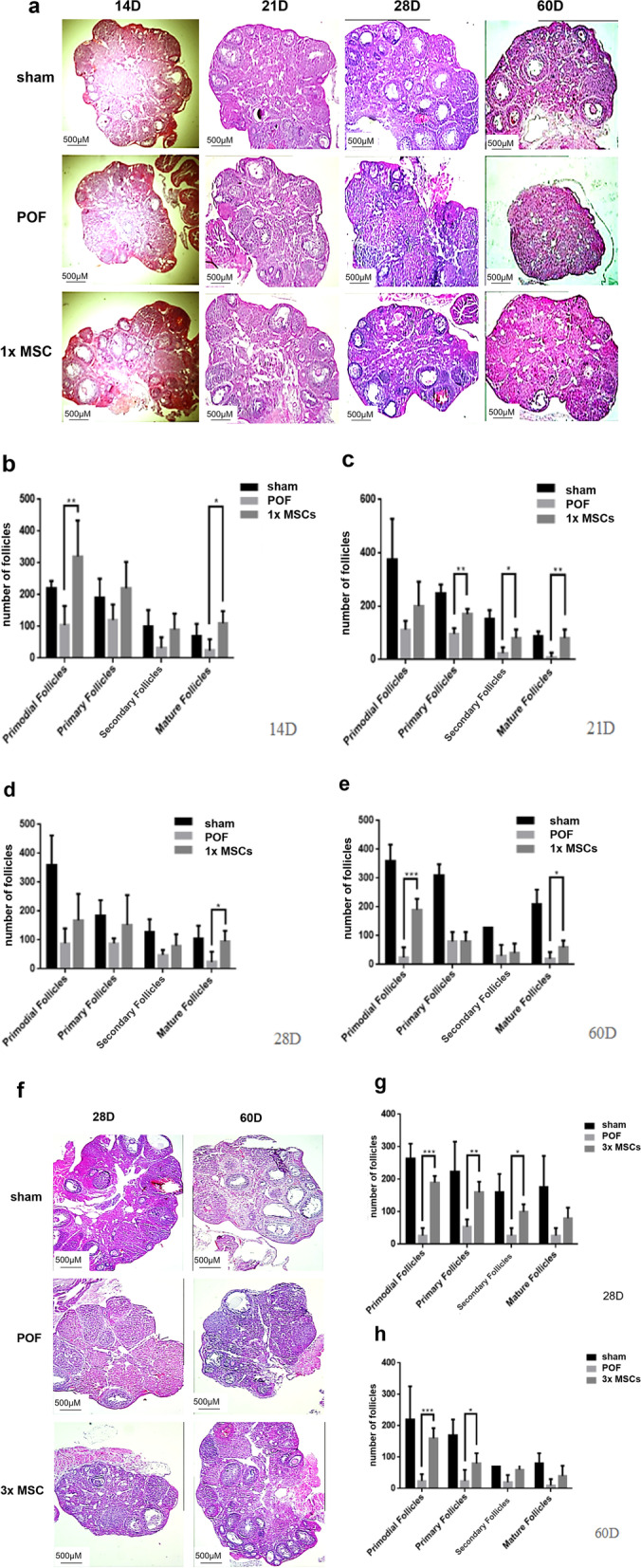
The effects of hUC-MSC transplantation on ovarian morphology and follicular development in the POF mouse. **a** Representative H&E micrographs of ovary sections from the once intravenous group over 60 days showing all stages of follicles in Sham group, stroma, and atretic primordial or primary follicles in POF group, primordial as well as primary and large antral follicles in MSCs group. *n* = 5. Original magnification: 100 × . **b** Follicle counts at all stages on the 14th day. *n* = 5. **c** Follicle counts at all stages on the 21th day. *n* = 5. **d** Follicle counts at all stages on the 28th day. *n* = 5. **e** Follicle counts at all stages on the 60th day. *n* = 5. **f** Ovarian sections of the multiple intravenous injection group. Original magnification: 100 × . *n* = 5. **g** Morphological follicle count of mouse ovaries, the follicle counts at all stages on the 28th day. *n* = 5. **h** Morphological follicle count of mouse ovaries, the follicle counts at all stages on the 60th day. *n* = 5. *P < 0.05; **P < 0.01; ***P < 0.001. 1xMSC: Single transplantation of hUC-MSC; 3xMSC: Multiple transplantation of hUC-MSC

We observed the number and development of follicles at all stages in the ovaries by H&E staining (Fig. 5a and f). In the sham group, well-developed primordial, primary, secondary, and mature follicles were observed. In the POF mouse model, the ovaries were atrophied, the number of follicles at each stage decreased, and the number of atretic follicles was high. In the single hUC-MSC injection group, the number of mature follicles increased significantly compared with the POF group. The number of follicles at various developmental stages also recovered significantly (*P* < 0.05, Fig. 5b–e). In the multiple hUC-MSC injection group, the number of primordial and primary follicles increased significantly (*P* < 0.05, Fig. 5g and h). The results showed that hUC-MSCs had a good therapeutic effect on follicle recovery. At D28, the number of primordial, primary, and secondary follicles in the hUC-MSC multiple transplantation group was significantly increased compared with that in the POF group; however, this phenomenon was not observed in the single hUC-MSC transplantation group. This suggested that multiple hUC-MSC transplantations have a better therapeutic effect on POF.

### hUC-MSC transplantation restored hormone levels

**Fig. 6:**
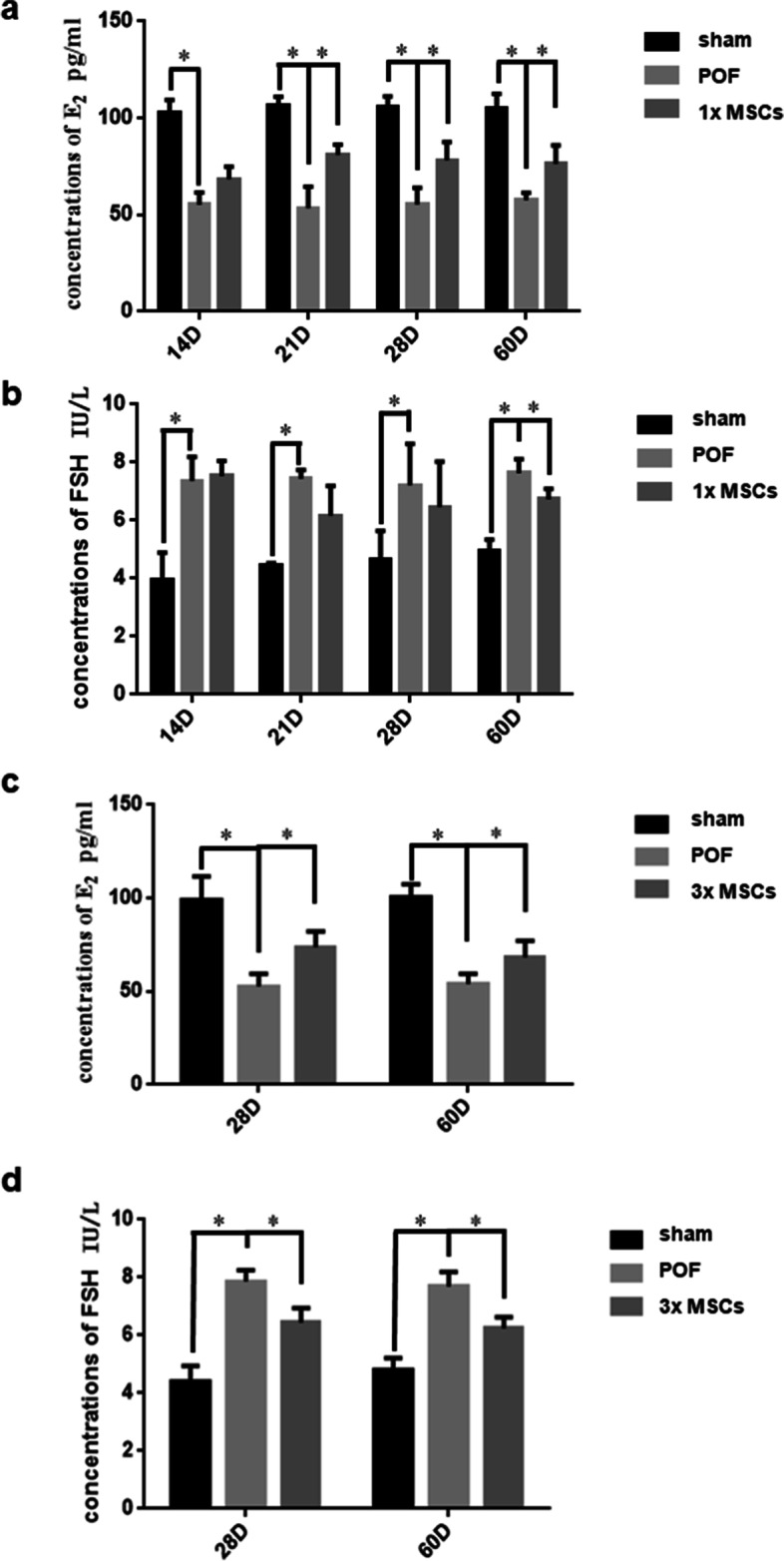
The effect of hUC-MSCs on the endocrine function of POF mice. **a** The hormone levels of E_2_ from the once intravenous group and the multiple intravenous group. Comparing the POF and MSCs group, the level of E_2_ had a significant increase at 21, 28, 60 days post-induction. *n* = 5. **b** The level of FSH from the once intravenous group and the multiple intravenous group. It had an obvious difference at D60. *n* = 5. **c** In the multiple group, the levels of E_2_ were significant differences at 28, 60 days post-induction. *n* = 5. **d** In the multiple group, the levels of FSH were significant differences at 28, 60 days post-induction. *n* = 5, *P < 0.05. 1xMSC: Single transplantation of hUC-MSC; 3xMSC: Multiple transplantation of hUC-MSC

To further study the therapeutic effect of hUC-MSCs on mouse POF, we used ELISA to determine the expression of E_2_ and FSH in mouse serum. As shown in Fig. 6, E_2_ expression in the POF group was significantly downregulated, and FSH expression was significantly upregulated compared with the sham group. Mouse serum E_2_ expression was upregulated in the single hUC-MSC transplantation group than in the POF group, and it was significantly upregulated on D21, D28, and D60 (*P* < 0.05, Fig. 6a). FSH expression decreased significantly on D60 (*P* < 0.05, Fig. 6c). Mice treated with multiple hUC-MSC transplantation showed a significant increase in serum E_2_ and FSH levels on D28 and D60 (both *P* < 0.05, Fig. 6b and d) relative to mice in the POF group. The results showed that the multiple hUC-MSC transplantation group performed better than the POF group, and FSH recovery in serum was more evident at D28, superior to the single hUC-MSC transplantation group. Multiple hUC-MSC transplantations may have a better therapeutic effect on POF.

### hUC-MSCs upregulate the expression of related molecules in ovarian granulosa cells

**Fig. 7:**
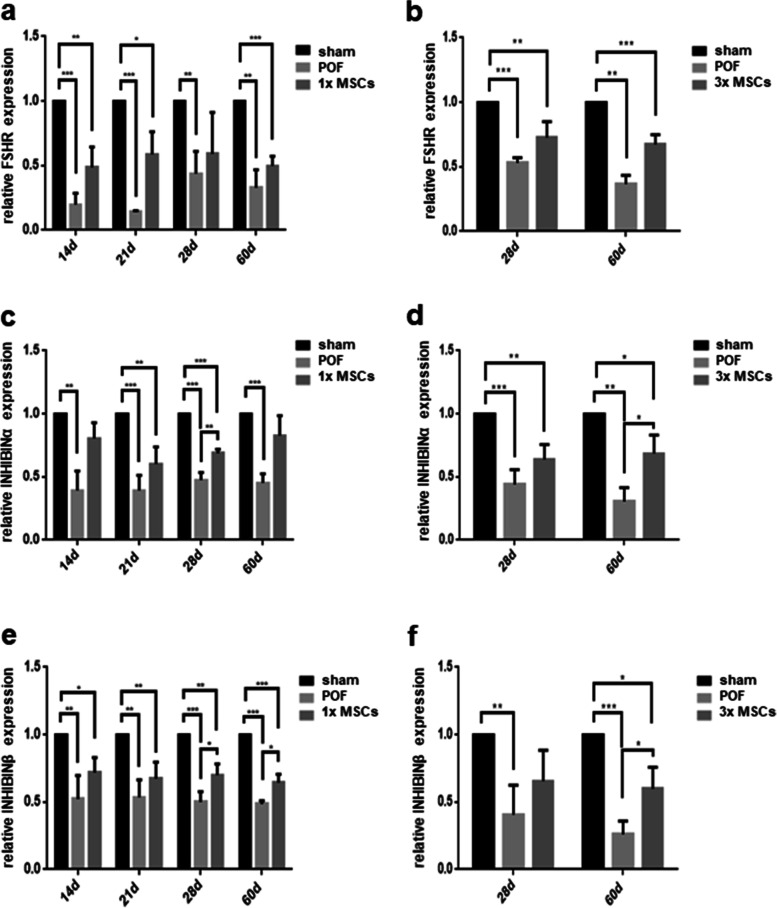
Effect of hUC-MSCs on the ovarian gene expression of POF mice. **a** Expression of FSHR in mouse ovary in single injection of hUC-MSCs group. *n* = 5. **b** Expression of FSHR in mouse ovary in the hUC-MSCs group injected multiple times. *n* = 5. **c** Expression of INHIBINα in mouse ovary in single injection of hUC-MSCs group. *n* = 5. **d** Expression of INHIBINα in mouse ovary in the hUC-MSCs group injected multiple times. *n* = 5. **e** Expression of INHIBINβ in mouse ovary in single injection of hUC-MSCs group. *n* = 5. **f** Expression of INHIBINβ in mouse ovary in the hUC-MSCs group injected multiple times. *n* = 5. *P < 0.05; **P < 0.01; ***P < 0.001. 1xMSC: Single transplantation of hUC-MSC; 3xMSC: Multiple transplantation of hUC-MSC

We selected the genes *FSHR*, *INHIBINα*, and *INHIBINβ*, which mainly exist in the granulosa cells of follicles, and detected the changes in gene expression using qRT-PCR. The therapeutic effect of hUC-MSCs on POF was tested, and the results are shown in Fig. 7. The results showed that the expression of *FSHR*, *INHIBINα*, and *INHIBINβ* in the POF group was significantly downregulated compared with that in the sham group (*P* < 0.01). *FSHR* expression was more upregulated in the single hUC-MSC transplantation group than in the POF group (Fig. 7a); *INHIBINα* and *INHIBINβ* expression was significantly upregulated on D28, and D28 and D60, respectively (Fig. 7c, *P* < 0.01 and Fig. 7e, *P* < 0.05, respectively). *FSHR* expression was significantly upregulated in the multiple hUC-MSC injection group compared with the POF group (Fig. 7b); *INHIBINα* and *INHIBINβ* expression was significantly upregulated on D60 (Fig. 7d and f, respectively, both *P* < 0.05). hUC-MSC transplantation increased the expression of *FSHR*, *INHIBINα*, and *INHIBINβ* in ovarian tissues compared with the POF group. Multiple hUC-MSC injections had a more significant upregulation effect on *INHIBINα* and *INHIBINβ* on D60 (*P* < 0.05), showing a better therapeutic effect.

**Fig. 8:**
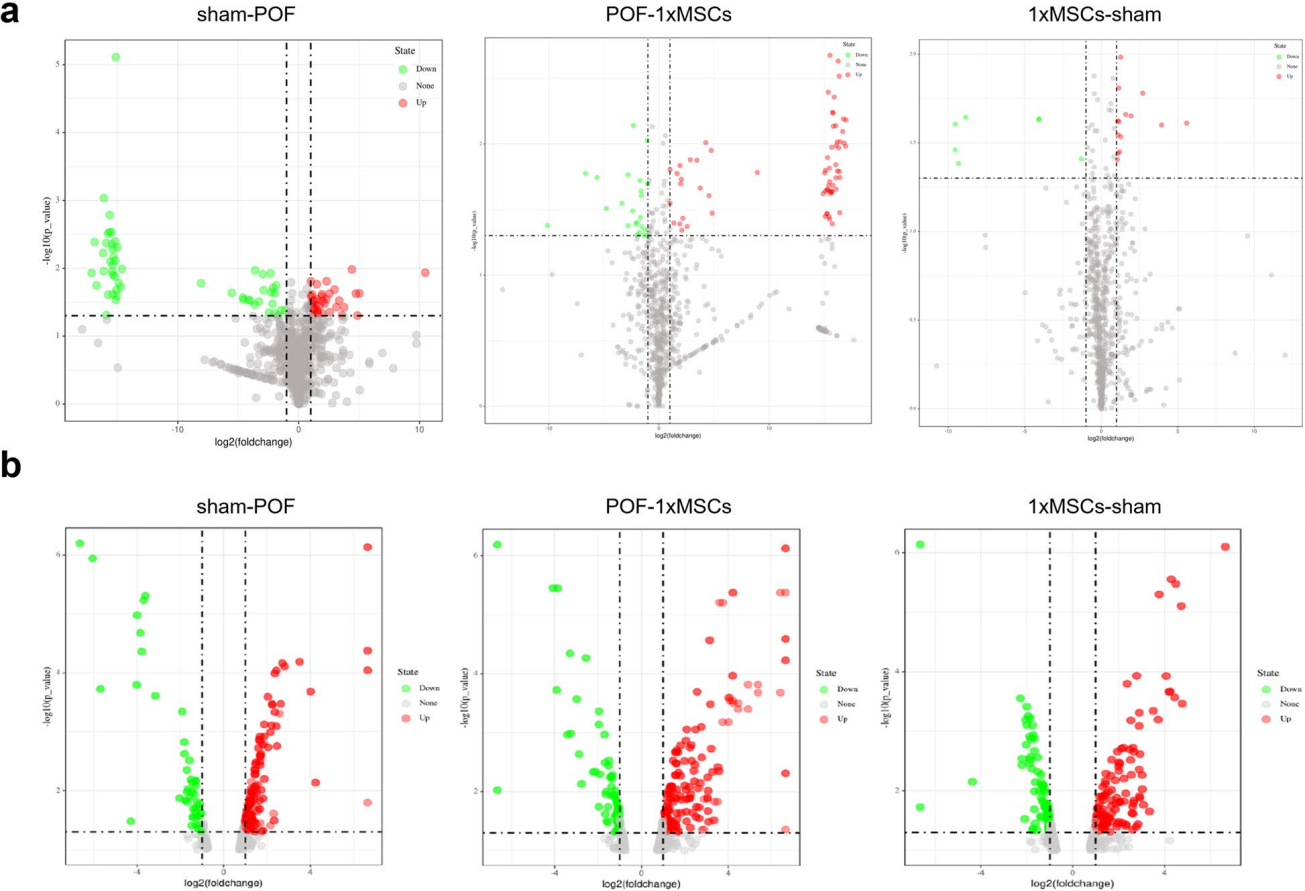
miRNA chip and mRNA chip detection of mouse ovaries. **a** Volcano map to detect differentially expressed miRNAs between different treatment groups. **b** Volcano map analysis of the mRNA chip. The ovarian tissue in hUC-MSC group was got from mice 7 days after first injection hUC-MSCs by tail vein

We used mRNA and miRNA chips to screen differentially expressed genes in ovarian tissue 7 d after hUC-MSC transplantation. Volcano mapping is a common method for observing differentially expressed genes between two groups. It can visually illustrate the fold-change and significance of the difference in the relationship between genes. Figure 8a shows the miRNA chip volcano graph analysis of the POF and 1 × MSC treatment groups, the sham operation and 1 × MSC treatment groups, and the POF and sham operation groups. Figure 8b shows a volcano map analysis of the mRNA chip where green indicates downregulated genes and red indicates upregulated genes. We observed that the miRNA chip showed fewer differential genes between the sham and 1 × MSC treatment groups, indicating that 1 × MSC repaired chemotherapy-induced POF damage at the gene level. We screened 18 miRNAs and 55 mRNAs that were upregulated after POF and downregulated after 1 × MSC treatment (Table 1); 38 miRNAs and 21 mRNAs were downregulated after POF and upregulated after 1 × MSC treatment (Table 2).

**Table 1:** The expression of premature ovarian failure is up-regulated, and the expression of differentially expressed genes is down-regulated after hUC-MSCs treatment

Name	POF/SHAM UP		MSCs/POF DOWN		MSCs/SHAM	
	Fold change values	*P* value	Fold change values	*P* value	Fold change values	*P* value
mmu-miR-101c	1.93	0.024	-2.11	0.040	-0.19	0.349
mmu-miR-1892	1.99	0.030	-1.36	0.049	0.64	0.057
mmu-miR-1894-3p	1.52	0.045	-1.03	0.020	0.49	0.141
mmu-miR-3070–2-3p	5.03	0.023	-5.62	0.018	-0.59	0.162
mmu-miR-3072-5p	2.97	0.020	-1.99	0.039	0.98	0.101
mmu-miR-3102-5p.2-5p	3.35	0.029	-2.39	0.032	0.96	0.119
mmu-miR-328-5p	1.56	0.042	-1.58	0.042	-0.01	0.904
mmu-miR-3547-5p	1.53	0.028	-1.05	0.049	0.48	0.138
mmu-miR-363-5p	2.56	0.044	-6.65	0.016	-4.09	0.023
mmu-miR-374b-5p	0.32	0.024	-0.60	0.007	-0.28	0.027
mmu-miR-374c-5p	0.40	0.042	-0.86	0.018	-0.46	0.016
mmu-miR-5122	1.05	0.033	-0.99	0.044	0.06	0.545
mmu-miR-652-5p	1.01	0.015	-0.99	0.020	0.03	0.610
mmu-miR-6965-5p	4.40	0.010	-2.80	0.017	1.60	0.021
mmu-miR-7088-5p	1.28	0.040	-0.61	0.033	0.67	0.076
mmu-miR-7235-5p	0.85	0.041	-0.66	0.042	0.19	0.192
mmu-miR-7648-5p	4.73	0.023	-2.80	0.041	1.94	0.079
mmu-miR-8110	1.51	0.017	-0.86	0.033	0.65	0.020
apoptosis-associated tyrosine kinase (Aatk)	1.10	0.022	-1.66	0.002	-0.55	0.248
apolipoprotein F (ApoF)	0.91	0.051	-2.27	0.002	-1.38	0.011
beta-1,3-N-acetylgalactosaminyltransferase 1 (B3galnt1)	0.73	0.099	-0.82	0.071	-0.04	0.914
apoptosis repeat containing 5 (Birc5)	0.74	0.090	-0.86	0.051	-0.13	0.740
calmodulin binding transcription activator 1 (Camta1)	0.93	0.040	-0.96	0.036	-0.01	0.997
coiled-coil domain containing 144B (Ccdc144b)	0.89	0.080	-1.05	0.044	-0.16	0.748
cytidine/uridine monophosphate kinase 2 (Cmpk2)	1.42	0.004	-1.53	0.002	-0.08	0.848
cytochrome C oxidase Subunit 7A1 (Cox7a1)	0.80	0.083	-0.89	0.057	-0.07	0.865
cathepsin K (Ctsk)	1.68	0.003	-1.06	0.038	0.64	0.239
c-x-c motif chemokine ligand 9 (Cxcl9)	1.50	0.036	-0.73	0.252	0.85	0.281
DExD/H-Box helicase 60 (Ddx60)	1.86	0.001	-1.09	0.026	0.81	0.116
DExD/H-Box helicase 58 (Dhx58)	1.49	0.008	-1.14	0.031	0.37	0.502
Eukaryotic translation initiation factor 2 alpha kinase 2 (Eif2ak2)	1.14	0.018	-0.92	0.049	0.24	0.590
family with sequence similarity 78 member A (Fam78a)	0.79	0.076	-1.03	0.025	-0.21	0.618
Fc fragment of IgG receptor Ia (Fcgr1)	2.32	0.032	-1.80	0.074	0.68	0.667
guanylate binding protein 3 (Gbp3)	1.66	0.001	-1.21	0.010	0.45	0.297
predicted gene 12250 (Gm12250)	1.87	0.006	-1.05	0.072	0.90	0.207
predicted gene 4951 (Gm4951)	1.81	0.013	-1.16	0.075	0.74	0.365
H2A clustered histone 6 (Hist1h2ao)	1.06	0.021	-0.73	0.096	0.33	0.432
integrin binding sialoprotein (Ibsp)	3.50	0.000	-1.46	0.009	2.19	0.008
interferon alpha inducible protein 27 (Ifi27l2a)	2.03	0.000	-1.75	0.000	0.26	0.525
interferon induced protein 44 (Ifi44)	2.63	0.000	-2.02	0.001	0.68	0.329
interferon induced protein 47 (Ifi47)	1.43	0.005	-0.83	0.072	0.62	0.187
interferon induced with helicase C domain 1 (Ifih1)	1.43	0.049	-1.31	0.069	0.16	0.848
interferon induced protein with tetratricopeptide repeats 1 (Ifit1)	2.70	0.000	-2.05	0.000	0.69	0.282
interferon induced protein with tetratricopeptide repeats 2 (Ifit2)	0.87	0.068	-1.01	0.038	-0.13	0.776
immunity related GTPase (Igtp)	1.46	0.004	-0.94	0.042	0.53	0.251
interferon regulatory factor 7 (Irf7)	2.42	0.000	-2.00	0.000	0.43	0.344
interferon regulatory factor 9 Irf9	0.83	0.069	-0.81	0.076	0.04	0.925
immunity related GTPase 1 (Irgm1)	1.41	0.008	-0.93	0.059	0.51	0.320
immunity related GTPase 2 (Irgm2)	1.22	0.014	-0.78	0.091	0.45	0.329
ISG15 ubiquitin like modifier (Isg15)	2.42	0.000	-1.89	0.003	0.61	0.401
inositol 1,4,5-trisphosphate receptor type 2 (Itpr2)	0.72	0.098	-0.82	0.064	-0.10	0.810
LEO1 Homolog (Leo1)	1.09	0.026	-1.08	0.028	0.03	0.946
galectin 3 binding protein (Lgals3bp)	1.63	0.001	-1.04	0.021	0.58	0.176
galectin 9 (Lgals9)	0.90	0.049	-0.78	0.083	0.15	0.727
ligand of numb-protein X 1 (Lnx1)	1.38	0.011	-0.85	0.087	0.55	0.300
LY6/PLAUR domain containing 2 (Lypd2)	6.64	0.000	-6.64	0.000	-0.03	0.950
MX dynamin like GTPase 2 (Mx2)	2.16	0.001	-1.85	0.002	0.35	0.575
out at first homolog (Oaf)	1.01	0.030	-0.93	0.043	0.10	0.808
2'-5'-oligoadenylate synthetase 2 (Oas2)	2.24	0.000	-2.00	0.000	0.25	0.641
2'-5'-oligoadenylate synthetase 1 (Oasl1)	1.65	0.014	-1.10	0.074	0.63	0.385
2'-5'-oligoadenylate synthetase 2 (Oasl2)	1.59	0.001	-1.23	0.009	0.37	0.387
poly (ADP-ribose) polymerase family member 14 (Parp14)	1.20	0.013	-0.92	0.044	0.30	0.501
poly(ADP-ribose) polymerase family member 9 (Parp9)	1.18	0.014	-0.92	0.045	0.28	0.526
placenta associated 8 (Plac8)	1.68	0.001	-1.11	0.016	0.57	0.184
RNA binding motif protein 22 (Rbm22)	0.91	0.046	-0.74	0.097	0.19	0.647
regulator of G protein Signaling 13 (Rgs13)	1.14	0.032	-1.85	0.002	-0.70	0.237
radical S-adenosyl methionine domain containing 2 (Rsad2)	2.20	0.000	-1.37	0.013	0.87	0.151
receptor transporter protein 4 (Rtp4)	2.36	0.000	-1.86	0.000	0.51	0.249
Schlafen Family Member 5 (Slfn5)	1.45	0.005	-1.09	0.024	0.38	0.421
Sarcalumenin (Srl)	4.24	0.007	-4.36	0.007	-0.12	0.803
tripartite motif containing 34A (Trim34a)	1.79	0.001	-1.51	0.004	0.29	0.567
ubiquitin specific peptidase 18 (Usp18)	2.23	0.000	-1.82	0.001	0.42	0.419
zinc finger protein 385B (Zfp385b)	0.86	0.054	-1.02	0.026	-0.15	0.711

**Table 2:** The expression of premature ovarian failure is down-regulated, and the differentially expressed genes are up-regulated after hUC-MSCs treatment

Name	POF/Sham DOWN		MSCs/POF UP		MSCs/SHAM	
	Fold change values	*P* value	Fold change values	*P* value	Fold change values	*P* value
mmu-let-7c-1-3p	-15.41	0.005	16.37	0.016	0.96	0.284
mmu-miR-125b-2-3p	-2.12	0.019	2.17	0.037	0.05	0.779
mmu-miR-148b-5p	-16.18	0.005	16.95	0.010	0.77	0.254
mmu-miR-152-5p	-15.20	0.013	16.27	0.002	1.08	0.177
mmu-miR-1946b	-15.44	0.004	15.68	0.023	0.24	0.855
mmu-miR-200a-3p	-0.77	0.049	2.04	0.019	1.27	0.010
mmu-miR-200b-3p	-0.77	0.036	1.91	0.015	1.14	0.016
mmu-miR-221-5p	-16.11	0.001	16.75	0.006	0.64	0.168
mmu-miR-224-5p	-16.88	0.004	16.81	0.010	-0.07	0.815
mmu-miR-26a-2-3p	-15.77	0.003	15.57	0.024	-0.20	0.691
mmu-miR-26b-3p	-15.36	0.012	15.44	0.036	0.09	0.915
mmu-miR-27b-5p	-15.91	0.004	16.42	0.018	0.51	0.521
mmu-miR-3084-5p	-2.16	0.033	1.89	0.041	-0.26	0.153
mmu-miR-342-5p	-15.38	0.009	16.08	0.022	0.70	0.493
mmu-miR-34c-5p	-0.25	0.044	0.51	0.019	0.26	0.031
mmu-miR-3535	-0.77	0.047	0.91	0.007	0.14	0.247
mmu-miR-378a-5p	-8.08	0.017	8.93	0.017	0.85	0.119
mmu-miR-381-5p	-16.72	0.018	15.09	0.036	-1.63	0.313
mmu-miR-410-3p	-1.99	0.023	1.35	0.040	-0.64	0.080
mmu-miR-433-3p	-4.57	0.029	4.25	0.010	-0.31	0.391
mmu-miR-466f-3p	-1.12	0.042	1.01	0.028	-0.10	0.439
mmu-miR-487b-3p	-3.29	0.439	2.85	0.013	-0.45	0.000
mmu-miR-495-3p	-4.18	0.029	3.75	0.021	-0.43	0.248
mmu-miR-505-3p	-15.43	0.011	15.27	0.022	-0.16	0.767
mmu-miR-5107-3p	-14.88	0.017	16.05	0.014	1.17	0.269
mmu-miR-6540-3p	-15.16	0.008	15.80	0.021	0.63	0.491
mmu-miR-669a-3-3p	-2.28	0.023	1.98	0.020	-0.30	0.133
mmu-miR-669b-3p	-14.72	0.019	15.45	0.023	0.73	0.505
mmu-miR-669e-3p	-4.61	0.027	4.83	0.034	0.21	0.429
mmu-miR-669 h-3p	-15.38	0.024	16.08	0.010	0.71	0.428
mmu-miR-677-5p	-16.12	0.011	16.05	0.007	-0.08	0.886
mmu-miR-7047-3p	-14.62	0.010	16.68	0.008	2.05	0.096
mmu-miR-7056-5p	-2.89	0.021	2.08	0.046	-0.81	0.130
mmu-miR-706	-5.53	0.023	4.55	0.025	-0.98	0.084
mmu-miR-7064-3p	-15.00	0.005	16.42	0.033	1.43	0.396
mmu-miR-7661-3p	-15.63	0.002	16.98	0.007	1.35	0.084
mmu-miR-7669-5p	-15.48	0.003	15.75	0.006	0.28	0.336
mmu-miR-9-3p	-15.30	0.007	16.07	0.018	0.77	0.383
abhydrolase domain containing 1 (Abhd1)	-1.81	0.002	0.91	0.099	-0.89	0.074
cortexin 3 (Ctxn3)	-1.44	0.028	2.05	0.003	0.64	0.227
duffy antigen receptor for chemokines (Darc)	-1.69	0.010	1.46	0.026	-0.23	0.663
family with sequence similarity 83 member C (Fam83c)	-1.78	0.014	1.69	0.023	-0.07	0.891
FGGY carbohydrate kinase domain containing (Fggy)	-1.29	0.008	1.16	0.015	-0.12	0.765
glucosaminyl (N-acetyl) transferase 3 (Gcnt3)	-1.18	0.037	1.93	0.002	0.79	0.117
glycerophosphodiester phosphodiesterase domain containing 3 (Gdpd3)	-3.69	0.000	4.29	0.000	0.60	0.158
interleukin 4 receptor A(Il4ra)	-3.15	0.000	2.62	0.001	-0.50	0.278
myo-inositol oxygenase (Miox)	-0.77	0.086	0.78	0.086	0.02	0.958
P21 (RAC1) activated kinase 1 (Pak1)	-1.47	0.007	1.21	0.024	-0.24	0.600
phosphatidylinositol-4-phosphate 3-kinase catalytic subunit type 2 gamma (Pik3c2g)	-1.09	0.016	1.06	0.019	-0.05	0.901
protein tyrosine phosphatase receptor type A (Ptpra)	-0.84	0.084	0.88	0.072	0.07	0.864
regulator of G protein signaling 9 (Rgs9)	-3.78	0.000	2.88	0.001	-0.93	0.067
45S pre-ribosomal RNA (Rn45s)	-1.48	0.038	1.26	0.088	-0.20	0.734
secreted and transmembrane 1A (Sectm1a)	-3.99	0.000	2.52	0.001	-1.44	0.005
secreted and transmembrane 1B (Sectm1b)	-6.05	0.000	1.99	0.003	-4.08	0.000
stress associated endoplasmic reticulum protein family member 2 (Serp2)	-1.65	0.065	1.77	0.059	0.13	0.850
solute carrier family 15 member 2 (Slc15a2)	-1.47	0.010	1.65	0.005	0.20	0.665
transcobalamin 2 (Tcn2)	-0.93	0.040	1.10	0.019	0.17	0.678

## Discussion

The incidence of POF in women is 1% [27], and CTX treatment is among the causes of POF [28]. Therefore, treating degenerative ovarian changes and restoring ovarian function is key to ensuring female reproductive health [29]. Current methods of ovarian function restoration include ovarian transplantation [30–32], autologous mitochondrial microinjection [33], and dormant follicle activation [34], among others. However, problems, such as rejection reaction, oocyte quality decline, and surgical violations remain to be resolved. We chose MSCs to treat POF owing to their low immunogenicity [35] and excellent performance against degenerative diseases. MSCs can treat injured sites through homing and exocrine effects and reduce damage to the body caused by surgery. Nevertheless, because of the limited survival time of MSCs in the body, the number of MSCs required for each treatment and the number of treatments remain controversial [36].

The umbilical cord is considered waste after childbirth, but there is evidence that, compared to MSCs derived from classic cell sources, such as bone marrow or adipose tissue, UC-MSC shows special advantages, such as increased pluripotency and prolonged proliferation capacity [37]. Therefore, UC-MSC may be more suitable for cell therapy. hUC-MSCs have been shown to exhibit high differentiation potential and proliferative activity. Moreover, hUC-MSCs express the stem cell markers CD44, CD73, CD90, and CD105 [38–40] and have the potential to differentiate into cells of all three embryonic tissue layers [41–43]. MSCs are multipotent cells defined in part by their ability to generate adipose, osteogenic, and chondrogenic cell in vitro [44, 45]. We conducted experiments on the induction of osteogenic and adipogenic differentiation of stem cells to further verify that the isolated cells are hUC-MSCs.

In order to test whether hUC-MSCs play a role in the treatment of POF, we took samples of mouse ovaries 7 days after injection of hUC-MSCs to detect these with anti-human nuclear antibody. Anti-human nuclear primary antibody is specific because the positive staining of the human nuclei antigen was completely colocalized with the nuclei of hUC-MSCs. The human nuclei antigen was detected in the nucleus of the follicles of MSC-treated animals, and was also found in the non-nuclear ovarian tissue of MSC-treated animals. We speculated that they might be the lysed MSCs because allogeneic MSCs were susceptible to lysis by cytotoxic CD8( +) T and NK cells [46]. hUC-MSCs detected near the follicles suggest that hUC-MSCs can survive in mice and play a therapeutic role [47]. The homing of stem cells means that they can migrate directly to damaged tissues on impulse and survive there under the stimulation of multiple factors, which helps the ovaries to recover. Liu et al. demonstrated that MSCs return to the ovaries through blood circulation to restore the ovarian structure and function of POF model rats [48]. Gabr et al. showed that MSCs can locate and survive in the damaged ovary, thereby promoting the restoration of the histological structure and endocrine function of the ovary [49]. This result is consistent with the experimental results of Mohamed et al. [36] and Song et al. [50] found that hUC-MSCs can survive in vivo for 4 weeks. Therefore, we selected 60 days as the longest study time to test the therapeutic effect of hUC-MSCs. These results are consistent with those of Xiao [51] and Jaalie [52] et al. who found that MSCs may support the survival of ovarian follicles through paracrine.

Improvement in female sex hormone levels is an important indicator of ovarian function restoration. Hormonal imbalance with a low concentration of E2 and a high concentration of FSH in serum is a typical symptom of POF [53]. In the present study, 60 days after POF model induction, the E2 and FSH levels of mice injected multiple times with hUC-MSCs were restored, indicating that multiple transplantation of hUC-MSCs can improve the ovarian endocrine function damaged by chemotherapy drugs. In the fertility test, we found that the fertility of POF mice treated with hUC-MSCs was significantly restored on D14 and D60. The results showed that hUC-MSCs have a long-term treatment effect on the ovaries of POF mice and can significantly restore ovary fertility. To confirm hUC-MSC ovarian function improvement, we evaluated the ovary organ coefficient and the number of follicles. The ovary organ coefficient significantly increased in the multiple transplantation group compared with the POF group. After hUC-MSC transplantation, all follicle stages in the MSC group were more enhanced than those in the POF group. In the ovaries of chemotherapy-induced POF mice, there remained some primordial and primary follicles. Fourteen days after POF induction, the numbers of primitive and mature follicles increased significantly in the single hUC-MSC treatment group compared with the POF group. It is possible that hUC-MSCs temporarily prevent follicular atresia and maintain follicular development in the ovaries of POF mice [54]. AMH, secreted by follicular granulosa cells, is one of the best indicators of ovarian reserve [55]. We used immunohistochemistry to analyze the AMH that was specifically stained on antral follicles. The number of follicles at all stages in POF mice was lower than that in the sham operation group, but a small number of follicles were still produced. The results of AMH immunohistochemistry showed that the granulosa cells of antral follicles in the POF group had little AMH staining, suggesting that even if a small number of antral follicles were produced in the POF group, the ovarian function was still difficult to recover. Ki67 is a key factor in ribosome synthesis during cell division, which is necessary for cell proliferation [56]. The results of immunohistochemistry showed that the expression of AMH and Ki67 in the ovarian tissue of mice in the multiple hUC-MSC treatment group was significantly upregulated compared with the POF group at 28 and 60 days after POF induction. There was also an upregulation trend in the single hUC-MSC injection group, but there was no significant difference. It is possible that multiple transplantation of hUC-MSCs has a better recovery effect on the POF ovarian reserve.

To further verify the effect of hUC-MSCs on the restoration of ovarian function at the gene level, we used qRT-PCR to evaluate the expression of *FSHR*, *INHIBINα*, and *INHIBINβ*. The results showed that the expression in the hUC-MSC group was higher than that in the POF group. Under hUC-MSC treatment, ovarian granulosa cell and FSH secretion increased, restoring ovarian function. As the hUC-MSC treatment group promoted the expression of ovarian development-related factors compared with the POF group and significantly restored the number of follicles and organ coefficients, this suggests that hUC-MSCs has a therapeutic effect on POF. In order to further explore the molecular mechanism of hUC-MSCs repairing POF mice, we used miRNA and mRNA chips for screening. In miRNA chips, 18 miRNAs were found to be upregulated in POF, and their expression was restored after hUC-MSC treatment. At the same time, 38 downregulated miRNAs in POF were screened out, and their expression was restored after hUC-MSC treatment. In the mRNA chip, 55 mRNAs were upregulated in POF and recovered after hUC-MSC treatment. Overall, 21 mRNAs were downregulated in POF and recovered after hUC-MSC treatment. In summary, hUC-MSC transplantation can treat chemotherapy-induced POF. The organ coefficient and hormone level recovery effect in multiple transplantation mice were superior to those in single transplantation mice. However, further investigations are needed to confirm the mechanism involved in ovarian function recovery.

## Data Availability

The datasets generated during and/or analyzed during the current study are available from the corresponding author on reasonable request.
